# Anti-inflammatory/anti-fibrotic effects of the hepatoprotective silymarin and the schistosomicide praziquantel against *Schistosoma mansoni*-induced liver fibrosis

**DOI:** 10.1186/1756-3305-5-9

**Published:** 2012-01-11

**Authors:** Naglaa M El-Lakkany, Olfat A Hammam, Walaa H El-Maadawy, Afkar A Badawy, Afaf A Ain-Shoka, Fatma A Ebeid

**Affiliations:** 1 Department of Pharmacology, Theodor Bilharz Research Institute, Warrak El-Hadar, Imbaba, P.O Box 30, Giza 12411, Egypt; 2 Department of Pathology, Theodor Bilharz Research Institute, Warrak El-Hadar, Imbaba, P.O Box 30, Giza 12411, Egypt; 3 Department of Pharmacology, Faculty of Pharmacy, Cairo University, Kasr El-Aini St., Cairo 11562, Egypt

**Keywords:** *Schistosoma mansoni*, silymarin, praziquantel, liver fibrosis, hydroxyproline, transforming growth factor-β1, matrix metalloproteinase-2, mast cells

## Abstract

**Background:**

Praziquantel (PZQ) is an isoquinoline derivative (2-cyclohexylcarbonyl-1, 2, 3, 6, 7, 11b-hexahydro-4H-pyrazino{2,1-a}-isoquinoline-4-one), and is currently the drug of choice for all forms of schistosomiasis. Silymarin, a standardized milk thistle extract, of which silibinin is the main component, is known for its hepatoprotective, anti-inflammatory, antioxidant activities, and hepatocyte regeneration. This study investigates the anti-inflammatory/anti-fibrotic effects of silymarin and/or PZQ on schistosomal hepatic fibrosis.

**Methods:**

*Schistosoma mansoni*-infected mice were divided into two large groups (I & II), each with four subgroups and were run in parallel. (i) Infected untreated; (ii) treated with silymarin, starting from the 4^th ^(3 weeks before PZQ therapy) or 12^th ^(5 weeks after PZQ therapy) weeks post infection (PI); (iii) treated with PZQ in the 7^th ^week PI; and (iv) treated with silymarin, as group (ii) plus PZQ as group (iii). Comparable groups of uninfected mice run in parallel with the infected groups. Mice of groups I and II were killed 10 and 18 weeks PI, respectively. Hepatic content of hydroxyproline (HYP), serum levels and tissue expression of matrix metalloproteinase-2 (MMP-2), transforming growth factor-β1 (TGF-β1) and number of mast cells were determined. In addition, parasitological, biochemical and histological parameters that reflect disease severity and morbidity were examined.

**Results:**

Silymarin caused a partial decrease in worm burden; hepatic tissue egg load, with an increase in percentage of dead eggs; modulation of granuloma size, with significant reduction of hepatic HYP content; tissue expression of MMP-2, TGF-β1; number of mast cells, with conservation of hepatic reduced glutathione (GSH). PZQ produced complete eradication of worms, eggs and alleviated liver inflammation and fibrosis. The best results were obtained, in most parameters studied, in groups of mice treated with silymarin in addition to PZQ.

**Conclusions:**

Our results point to silymarin as a promising anti-inflammatory and anti-fibrotic agent; it could be introduced as a therapeutic tool with PZQ in the treatment of schistosomal liver fibrosis, but further studies on mechanisms of silymarin and PZQ in chronic liver diseases may shed light on developing therapeutic methods in clinical practice.

## Background

Schistosomiasis caused by *S. mansoni *continues to be an important cause of parasitic morbidity and mortality worldwide and is the most common fibrotic disease to arise due to inflammation and the deposition of scar tissue around parasite eggs trapped in the liver [1]. It is usually characterized by an unnoticed acute phase, followed by liver fibrosis at chronic and advanced stages [2]. In fibrosis, an excessive deposition of extracellular matrix (ECM) components is observed, such as type III collagen in the fibrotic process, and types I and II collagen, fibronectin and proteoglycan at various stages of granuloma formation [3]. The activated hepatic stellate cells (HSCs) have now been identified as the primary source of extracellular matrix synthesis in liver fibrogenesis [4]. Fibrogenic cytokines, like transforming growth factor-β (TGF-β) are among the major cytokines involved in the activation process, causing enhanced proliferation of HSCs and matrix synthesis [5]. Mast cell hyperplasia in the liver has also been observed in a variety of experimental models of rat-liver fibrosis, such as that induced by CCl_4_, diethylnitrosamine, radiation, porcine serum, and bile duct resection [6]. Mast cells, which are derived from hematopoietic progenitors, leave the bone marrow and migrate to areas of inflammation. TGF-β1 is the most potent mast cell chemo-attractant and is responsible for this directional migration at femtomolar (fM) concentrations, and tissue maturation of mast cells [7]. Thus, the activation of mast cells and the subsequent exocytosis of granules are followed by production and secretion of cytokines and other factors that lead to leukocyte infiltration and local inflammation [8]. Matrix metalloproteinases (MMPs) are the major enzymes that degrade the various types of collagen. In the liver, MMP-2 is produced abundantly by the activated HSCs and fibroblasts, although other resident liver cells may be minor producers of MMP-2

[9]. It is well known that fibrosis is reversible whereas cirrhosis is irreversible, so it is important to prevent fibrosis progressing to cirrhosis. However, there is no ideal anti-fibrotic drug to date. Available therapies for many chronic liver diseases are ineffective, with liver transplantation as the only option. Novel approaches that attack the scarring response are therefore urgently needed [10]. Praziquantel (PZQ), as a safe anti-schistosome drug, has been used for more than 30 years [11]. In general, specific treatment of schistosomiasis results in parasite elimination, and later on, a slight reduction in hepatic fibrosis that is attributed to parasite eradication [12]. Silymarin, a standardized extract of the milk thistle (*Silybum marianum *[L.] Gaertner) has a long tradition as an herbal remedy; it was introduced as a "hepatoprotective" agent more than 30 years ago and used clinically in Europe and Asia for the treatment of liver diseases [13,14]. Silymarin consists of four flavonolignan isomers namely: silybin (also known as silybinin or silibinin), isosilybin, silydianin and silychristin [13]. Silybin is the most prevalent and biologically active of the four isomers and represents about 60-70% of silymarin, followed by silychristin (20%), silidianin (10%), and isosilybin (5%). Silymarin offers good protection in various toxic models of experimental liver diseases in laboratory animals. The protective action of silymarin is explicable in terms of its capacity for trapping free radicals and has a stabilizing effect on the cytoplasmic membranes. It promotes protein synthesis, helps in regenerating liver tissue, enhances glucuronidation and protects against glutathione depletion. Moreover, it is a potent anti-inflammatory, immunomodulatory and anti-fibrogenic agent in the liver [8,15]. In a recent study by Mata-Santos, *et al. *[16] on the effect of silymarin treatment in experimental schistosomiasis, silymarin did not affect parasite oviposition capacity; reduced granulomatous peri-ovular reaction in the liver, and decreased hepatic fibrosis in this infection. Since research into the effect of silymarin on schistosomal hepatic fibrosis is very limited, we are interested to test its anti-inflammatory/anti-fibrotic effects alone and with the anti-schistosomal PZQ, on acute and chronic schistosomal liver fibrosis. Parasitological criteria, function and histopathology of the liver, serum and tissue markers of liver fibrosis were also examined.

## Methods

### Drugs and dosage

Silymarin (Legalon^®^) purchased from (Chemical Industries Development (CID), Giza, Egypt under License of: Madaus GmbH. Germany) was given orally in a dose of 750 mg/kg/day [17], 5 days/week for 6 weeks in the form of aqueous suspension in 2% Cremophor El (Sigma Chemical Co., St. Louis, MO, USA). Praziquantel^® ^(Praziquantel-Sedico Pharmaceutical Co. 6^th ^of October City, Egypt) was given orally in a total dose of 1000 mg/kg divided equally on two consecutive days [18] in the form of aqueous suspension in 2% Cremophor El.

### Animals

Eighty CD-1 Swiss male albino mice, weighing 18-20 g were provided by the Schistosome Biology Supply Center (SBSC) of the Theodor Bilharz Research Institute (TBRI), Giza, Egypt. The mice were maintained on a standard commercial pelleted diet (El-Kahira company for oils and soap) in an air-conditioned animal house at 20-22^°^C. The animal experiments were conducted at the TBRI animal unit in accordance with international, ethical guidelines after approval of the institutional ethical committee of TBRI.

### Infection of animals

Animals were infected with the Egyptian strain of *S. mansoni *(70 ± 5 cercariae/mouse) using the body immersion technique according to the method described by Liang *et al. *[19].

### Experimental design

Experiment I (acute infection): *S. mansoni*-infected mice were divided into 4 groups, **Group (i) **infected mice received the vehicle (2% Cremophor El), **Group (ii) **treated with silymarin starting from the 4^th ^week (3 weeks before PZQ therapy) to the 10^th ^week PI. **Group (iii) **treated with PZQ in the 7^th ^week PI and **Group (iv) **treated with both silymarin and PZQ at the same time intervals as described in **Groups (ii) **and **(iii)**.

Experiment II (chronic infection): *S. mansoni*-infected mice were divided into 4 groups, **Group (i) **infected mice received the vehicle (2% Cremophor El), **Group (ii) **treated with silymarin starting from the 12^th ^week (5 weeks after PZQ therapy) to the 18^th ^week PI. **Group (iii) **treated with PZQ in the 7^th ^week PI and **Group (iv) **treated with both silymarin and PZQ at the same time intervals as described in **Groups (ii) **and **(iii)**. Animals of experiments I and II were killed by decapitation 10 and 18 weeks PI, respectively. Comparable groups of uninfected mice received the vehicle (2% Cremophor El) and were run in parallel with the infected groups and killed at a time corresponding to 10 and 18 weeks PI, respectively.

After decapitation, blood was collected and sera were separated by centrifugation at 1850 g for 10 minutes and stored frozen at -70°C prior to the pending assay. The livers were immediately chilled on ice and a piece of 0.5 g was homogenized using a Potter glass homogenizer equipped with a teflon pestle in 2.5 volumes (w/v) ice cold 0.1 M potassium phosphate buffer adjusted to pH 6.5. The crude homogenate was then centrifuged at 10.000 g for one hour at 4^°^C and the supernatant was harvested and snap frozen at -80^°^C in Eppendorf vials for subsequent analysis of the hepatic content of reduced glutathione (GSH). Another gram of liver was homogenized in 5 volumes (w/v) of 0.9% cold normal saline (0.9% NaCl). The crude homogenate was harvested and stored frozen at -70^°^C in Eppendorf vials for subsequent analysis of the hepatic content of HYP.

### Assessment of parasitological criteria

Hepatic and portomesenteric vessels were perfused [20] to recover worms for subsequent counting. The number of ova per gram of liver or intestine tissues was counted [21]. Percentage of the different egg developmental stages (oogram pattern) was examined [22].

### Histopathology and granuloma measurement

Livers recovered from mice were fixed in 10% buffered formalin and processed to paraffin blocks. Sections (4 μm thick) were cut 250 μm away from the preceding sections to avoid measurement of the same granuloma. Five paraffin liver sections were prepared from each animal and stained with haematoxylin and eosin (H&E) and Masson trichrome stains. Measurements of the granuloma size were conducted on non-contiguous granulomas, each containing a single egg (with intact or degenerated miracidia), using an ocular micrometer. The mean diameter of each granuloma was calculated by measuring two diameters of the lesion at right angles to each other [23]. For each mouse, 40 granulomas were measured and associated hepatic histopathological changes were studied.

### Liver function tests

Concentrations of alanine aminotransferase (ALT) and albumin in the collected sera were estimated using the available commercial kits (Sentinel CH, Milan, Italy). The level of reduced glutathione was determined in liver homogenate according to the method described by Ellman [24]. Briefly, 0.5 ml homogenate was added to a tube with 0.5 ml of 10% trichloroacetic acid. The tubes were centrifugated at 3000 g for 10 min. A 0.2 ml aliquot of the resulting supernatant was added to a tube containing 5 ml of 0.1 M phosphate buffer and 0.1 ml of 5, 5'-dithio-bis-(2-nitro benzoic acid; DTNB) solution (Ellman's reagent) and the absorbance was measured at 412 nm. A standard graph was drawn using different concentrations of a standard GSH solution (1 mg/ml). With the help of the standard graph, the GSH contents in the liver homogenates of the experimental animals were calculated.

### Liver fibrosis markers

As an index of liver fibrosis, (a) hydroxyproline was estimated in the liver using the method of Woessner [25]. Briefly, 0.5 ml of 20% liver homogenate was digested in 1 ml of 6 mol/L HCl at 120°C for 8 hours. An aliquot of digested homogenate (25 ml) was added to 25 ml citrate-acetate buffer and finally 500 ml of chloramines-T-solution was added and the mixture was left at room temperature for 20 min. Then, 500 ml Ehrlich's solution was added and the mixture was incubated at 65^°^C for 15 minutes. After cooling for 10 minutes, the color that developed was measured spectrophotometrically at 550 nm. (b) serum matrix metalloproteinases type-2 (MMP-2) determined using an ELISA kit (Quantikine R&D Systems, Minneapolis, Minnesota, USA) and (c) serum transforming growth factor β1 (TGF-β1) was determined using an ELISA kit (IBL International, GmbH, Hamburg, Germany).

### Immunohistochemical procedure

The standard avidin-biotin immunoperoxidase technique was used [26]. Paraffin sections (5 μm thick) were cut on positively charged slides, dewaxed in xylene and hydrated in descending grades of ethanol. The endogenous peroxidase activity was quenched by incubation in 100% methanol with 3% hydrogen peroxide for 20 minutes. Antigen retrieval was performed by subjecting the sections, in citrate buffer (pH 7.0), to 15 minutes of microwaves at 700 W. Sections were incubated overnight at +4°C in a humid chamber with primary antimouse monoclonal antibodies against TGF-β1 and MMP-2 (Santa Cruz Biotechnology, California, USA). The antibodies were diluted 1:50 and 1:100 respectively, in phosphate buffer saline (PBS). After rinsing in PBS, the sections were incubated at room temperature for 15 minutes with biotinylated secondary anti-mouse antibody and after a further wash in PBS, the slides were incubated with an avidin-biotin complex horseradish peroxidase solution (DAKO, Glostrup, Denmark). After 10 minutes of incubation, the peroxidase reaction was developed using 0.01% hydrogen peroxide in 0.05% diaminobenzidine tetrahydrochloride (DAB). Sections were counterstained with Meyer's hematoxylin and dehydrated in ethanol prior to mounting. Liver sections with the primary antibody replaced with PBS, served as negative controls, while colonic cancer sections served as TGF-β1 and MMP-2-positive controls.

The liver sections were examined using a Zeiss light microscope (Oberkochen, Germany). The number of positively stained cells with the highest expression recorded within 10 successive fields (x400) was counted per section animal in a semi-quantitative way for both markers; the final value represented the mean of 80 readings per group. Zero percentage was given to unstained sections. TGF-β1 expression sites were examined intralobularly, in the periportal areas, in hepatocytes, and granuloma [27]. MMP-2 expression sites were examined in hepatocytes, Kupffer cells and endothelial cells lining sinusoids and granuloma [28].

### Number of mast cells in liver tissue

Toluidine blue staining for mast cells was performed by immersion of liver sections in 0.1% toluidine blue (Sigma, USA) for 3-5 minutes at room temperature [29]. Both intact and degranulated mast cells as well as the total number of mast cells was quantified in 10 successive selected, non-overlapping, high power fields (x400) of each liver section [30].

### Statistical analysis

Data was analysed using version 11.0 of the SPSS software package (SPSS Inc., Chicago, IL, USA), with the results expressed as means with standard errors. Group means were compared using unpaired Student's *t*-tests. A *P*-value of < 0.05 was considered indicative of a statistically significant difference.

## Results

### Parasitological studies

Treatment of infected mice with silymarin for 6 weeks starting from the 4^th ^and 12^th ^weeks PI resulted in a reduction in the total worm burden; recovered 10 and 18 weeks PI by 26.25% (*P *> 0.05) and 39.39% (*P *< 0.001) respectively when compared to the infected untreated group. Moreover, a significant reduction (*P *< 0.01) in the hepatic tissue egg load by 28.73% and 43.13%, accompanied with significant increase (*P *< 0.05, *P *< 0.01) in the percentage of dead eggs was observed 10 and 18 weeks PI, respectively. Administration of PZQ alone or with silymarin resulted in 97-100% worm and egg eradication with ~77% reduction in the hepatic tissue egg load with no significant difference recorded between the two groups (Table 1).

**Table 1:** Effect of silymarin with/without praziquantel on total worms, hepatic tissue egg load and % dead eggs 10 and 18 weeks post infection of mice with *S.mansoni*

Animal groups	Total worms		Hepatic tissue egg load × 10^3^		% dead eggs	
	
	10 wks	18 wks	10 wks	18 wks	10 wks	18 wks
**Infected (Vehicle)**	19.16 ± 2.18	20.28 ± 0.99	16.22 ± 1.40	27.08 ± 3.53	7.16 ± 0.94	6.87 ± 0.99

**Infected + PZQ**	‡‡‡ 0.80 ± 0.17 (95.82%)	‡‡‡ 0.50 ± 0.27 (97.53%)	‡‡‡ 4.00 ± 0.43 (75.34%)	‡‡‡ 7.64 ± 1.10 (71.79%)	‡‡‡ 100 ± 0.00	‡‡‡ 100 ± 0.00

**Infected + silymarin**	14.13 ± 2.96 (26.25%)	‡‡‡ 12.29 ± 1.21 (39.39%)	‡‡ 11.56 ± 0.68 (28.73%)	‡‡ 15.40 ± 0.72 (43.13%)	‡ 11.00 ± 0.97	‡‡‡ 39.29 ± 2.60

**Infected + PZQ + silymarin**	‡‡‡ 0.50 ± 0.50 (97.39%)	‡‡‡ 0.00 ± 0.00 (100%)	‡‡‡ 3.67 ± 0.60 (77.37%)	‡‡‡ 6.14 ± 0.76 (77.33%)	‡‡‡ 100 ± 0.00	‡‡‡ 100 ± 0.00

Data are presented as mean ± SEM (n = 8 in each group).

Mice were administered praziquantel (PZQ; 500 mg/kg/day for 2 days) in the 7^th ^week post infection (PI) and silymarin (750 mg/kg/day, 5 days/week for 6 weeks) at the 4^th ^and 12^th ^weeks PI for 6 weeks and were killed 10 and 18 weeks PI respectively. Numbers in parentheses indicate the percentage of reduction from infected (vehicle) group.

‡ Significantly different from infected (vehicle) group at *P *< 0.05, ‡‡ *P *< 0.01 and ‡‡‡ at *P *< 0.001.

### Histopathological changes

**Figure 1:**
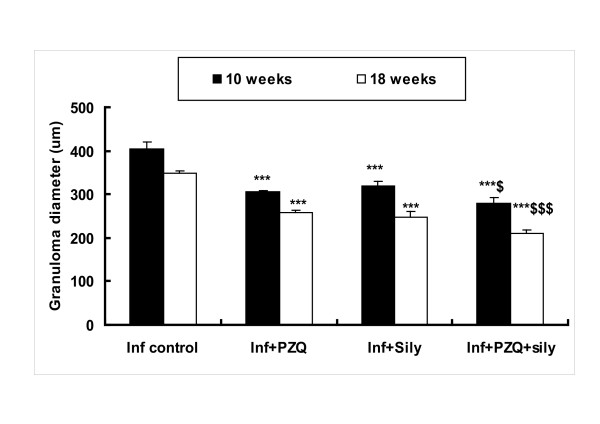
**Effect of silymarin with/without praziquantel on hepatic granuloma size, 10 and 18 weeks post-infection of mice with *S. mansoni***. *** significantly different from infected (vehicle) group at *P *< 0.001. $ significantly different from infected-PZQ treated group at *P *< 0.05, $$$ at *P *< 0.001.

Ten weeks post infection; the inflammatory granulomatous lesions were mainly seen in the hepatic parenchyma and to a lesser extent in the portal tracts. Fibrocellular granulomas, constituted around 60% of the estimated granulomas with mean granuloma diameter 403.83 ± 15.76 μm. In the chronic stage of *S. mansoni *infection (18 weeks PI) with immune modulation, fibrocellular granulomas (around 80% of the estimated granuloma) had fewer inflammatory cells and more fibrous tissue content with mean diameter of 349.50 ± 4.50 μm. With the progression of infection, the portal tracts were more expanded by the amalgamated granulomatous lesions that had developed around the newly deposited ova with subsequent collagen deposition along portal tracts. Treatment with PZQ reduced both the granuloma size and inflammatory cells 10 and 18 weeks PI. In addition, administration of silymarin to infected mice resulted in the regression of the granulomatous inflammatory reaction with significant reduction in granuloma size, 10 and 18 weeks PI when compared with their corresponding infected untreated groups. Administration of PZQ with silymarin resulted in a marked decrease in inflammatory cells in portal tracts and granulomas, with significant reduction in granuloma size (*P *< 0.05, *P *< 0.001), 10 and 18 weeks PI respectively, when compared to their corresponding PZQ treated groups (Figure 1).

### Liver function assessments

Infection of mice with *S. mansoni *showed a significant increase in serum levels of ALT (*P *< 0.01, *P *< 0.001) with a significant decrease in both albumin (*P *< 0.01) 18 weeks PI, and hepatic GSH (*P *< 0.001) 10 and 18 weeks PI in comparison with the uninfected untreated group. Compared to the infected untreated group, ALT level was significantly decreased in the group treated with either PZQ (*P *< 0.05, *P *< 0.01)) or silymarin (*P *< 0.05; 18 weeks PI) alone or with both drugs (*P *< 0.01, *P *< 0.001) 10 and 18 weeks PI, respectively. Moreover, serum albumin was significantly elevated in groups treated with either PZQ alone (*P *< 0.01) or combined with silymarin *(P *< 0.05) 18 weeks PI. In addition to that, the hepatic GSH was significantly elevated in groups treated with PZQ alone (*P *< 0.05*, P *< 0.001), silymarin alone (*P *< 0.01) or both (*P *< 0.01, *P *< 0.001), 10 and 18 weeks PI, respectively. Compared to the PZQ treated group, hepatic GSH was significantly restored in the group treated with both PZQ and silymarin 10 (*P *< 0.01) and 18 (*P *< 0.05) weeks PI (Table 2).

**Table 2:** Effect of silymarin with/without praziquantel on liver function tests, 10 and 18 weeks post-infection of mice with *S.mansoni*

Animal groups	Serum ALT (U/L)		Serum albumin (g/dl)		Hepatic GSH (mg/g liver)	
	
	10 wks	18 wks	10 wks	18 wks	10 wks	18 wks
**Uninfected (vehicle)**	22.62 ± 1.82	21.67 ± 0.93	3.75 ± 0.21	3.55 ± 0.23	6.00 ± 0.29	5.92 ± 0.26

**Infected (Vehicle)**	†† 40.71 ± 4.07	††† 32.57 ± 1.94	3.27 ± 0.10	†† 2.69 ± 0.07	††† 2.80 ± 0.22	††† 2.12 ± 0.21

**Infected + PZQ**	††‡ 30.57 ± 1.25	‡‡ 23.17 ± 1.76	3.35 ± 0.09	‡‡ 3.08 ± 0.07	†††‡ 3.40 ± 0.13	†††‡‡‡ 4.07 ± 0.29

**Infected + silymarin**	††† 39.75 ± 3.57	‡† 26.80 ± 2.00	3.22 ± 0.07	† 2.90 ± 0.05	††‡‡ 4.43 ± 0.32	†††‡‡ 3.18 ± 0.22

**Infected + PZQ + silymarin**	‡‡ 25.88 ± 1.41	‡‡‡ 19.75 ± 1.56	3.35 ± 0.12	‡ 3.17 ± 0.19	‡‡§§ 5.90 ± 0.68	†‡‡‡§ 4.99 ± 0.19

Data are presented as mean ± SEM (n = 8 in each group).

Mice were administered praziquantel (PZQ; 500 mg/kg/day for 2 days) in the 7^th ^week post infection (PI) and silymarin (750 mg/kg/day, 5 days/week for 6 weeks) at the 4^th ^and 12^th ^weeks PI for 6 weeks and were killed 10 and 18 weeks PI respectively.

ALT, Alanine aminotransferase; GSH, reduced glutathione.

† Significantly different from uninfected (vehicle) group at *P *< 0.05, †† at *P *< 0.01 and ††† at *P *< 0.001. ‡ Significantly different from infected (vehicle) group at *P *< 0.05, ‡‡ at *P *< 0.01 and ‡‡‡ at *P *< 0.001. § Significantly different from infected treated with PZQ group at *P *< 0.05 and §§ at *P *< 0.01.

### Liver fibrosis markers

Infection of mice with *S. mansoni *caused pronounced elevations in both serum TGF-β1 (*P *< 0.001) and MMP-2 (*P *< 0.01, *P *< 0.001) levels, 10 and 18 weeks PI respectively, when compared to their corresponding uninfected untreated groups. Compared to the infected untreated group, treatment with PZQ caused a significant reduction in both serum TGF-β1 *(P *< 0.001) and MMP-2 *(P *< 0.05*, P *< 0.001) levels by (43.34%, 51.75%) and (20.73%, 39.35%), 10 and 18 weeks PI, respectively. Treatment with silymarin resulted in a significant reduction of TGF-β1 (*P *< 0.05) by 32.73% 10 weeks PI with insignificant reductions (*P *> 0.05) in serum TGF-β1, 18 weeks PI and MMP-2 levels 10 and18 weeks PI. Administration of PZQ in combination with silymarin caused normalization with significant reduction of serum TGF-β1 (*P *< 0.01), 10 weeks PI and MMP-2 (*P *< 0.05), 10 and 18 weeks PI, when compared to the corresponding PZQ treated group (Table 3).

**Table 3:** Effect of silymarin with/without praziquantel on serum transforming growth factor-β1 and matrix metalloproteinase-2, 10 and 18 weeks post infection of mice with *S.mansoni*

Animal groups	Serum TGF-β1 (ng/ml)		Serum MMP-2 (ng/ml)	
	
	10 wks	18 wks	10 wks	18 wks
**Uninfected (vehicle)**	7.80 ± 0.69	7.98 ± 0.48	150.40 ± 18.17	181.00 ± 6.72

**Infected (Vehicle)**	††† 19.98 ± 2.37	††† 40.85 ± 1.41	†† 265.43 ± 21.65	††† 374.00 ± 32.16

**Infected+ PZQ**	‡‡‡ 11.32 ± 0.85 (43.34%)	†††‡‡‡ 19.71 ± 0.24 (51.75%)	††‡ 210.40 ± 8.13 (20.73%)	†††‡‡‡ 226.83 ± 11.69 (39.35%)

**Infected + silymarin**	†††‡ 13.44 ± 0.75 (32.73%)	††† 36.84 ± 1.44 (9.82%)	†† 241.31 ± 36.25 (9.093%)	††† 283.80 ± 42.20 (24.12%)

**Infected + PZQ + silymarin**	‡‡‡§§ 7.83 ± 0.65 (60.81%)	†††‡‡‡ 18.29 ± 0.73 (55.23%)	‡‡§ 165.33 ± 14.20 (37.71%)	‡‡‡§ 187.60 ± 11.57 (49.84%)

Data are presented as mean ± SEM (n = 8 in each group).

Mice were administered praziquantel (PZQ; 500 mg/kg/day for 2 days) in the 7^th ^week post infection (PI) and silymarin (750 mg/kg/day, 5 days/week for 6 weeks) at the 4^th ^and 12^th ^weeks PI for 6 weeks and were killed 10 and 18 weeks PI respectively. Numbers in parentheses indicate the percentage of reduction from infected (vehicle) group.

†† Significantly different from uninfected (vehicle) group at *P *< 0.01 and ††† at *P *< 0.001. ‡ Significantly different from infected (vehicle) group at *P *< 0.05, ‡‡ at *P *< 0.01 and ‡‡‡ at *P *< 0.001. § Significantly different from infected treated with PZQ group at *P *< 0.05 and §§ at *P *< 0.01.

Infection of mice with *S. mansoni *induced a significant increase (*P *< 0.001) in the hepatic content of HYP 10 and 18 weeks PI respectively, when compared to their corresponding uninfected untreated groups. Treatment with PZQ resulted in a significant reduction (*P *< 0.001) in HYP content by 34.25% and 39.61%, 10 and 18 weeks PI respectively, when compared to their corresponding infected untreated groups. In addition, treatment with silymarin showed significant reduction (*P *< 0.05) by 19.06% and 17.02% 10 and 18 weeks PI, respectively. Administration of PZQ with silymarin produced insignificant reduction in HYP content 10 and 18 weeks PI, when compared to their corresponding PZQ treated groups (Table 4).

**Table 4:** Effect of silymarin with/without praziquantel on hepatic hydroxyproline content and immunohistochemical expression of transforming growth factor-β1 and matrix metalloproteinase-2, 10 and 18 weeks post-infection of mice with *S.mansoni*

Animal groups	Within 10 successive microscopic fields (×400)/section/animal (Mean percentage +ve cells ± SEM)				Hydroxyproline content (μg/g liver)	
	
	TGF-β1		MMP-2			
	
	10 wks	18 wks	10 wks	18 wks	10 wks	18 wks
**Uninfected(vehicle)**	0.0 ± 0.0	0.0 ± 0.0	0.0 ± 0.0	0.0 ± 0.0	789.45 ± 50.61	763.82 ± 22.32

**Infected (Vehicle)**	50.60 ± 3.62	62.60 ± 4.21	37.00 ± 2.55	50.00 ± 5.70	††† 1495.88 ± 91.85	††† 1961.96 ± 80.97

**Infected + PZQ**	‡‡‡ 14.80 ± 0.37 (70.75%)	‡‡‡ 29.5 ± 2.01 (52.88%)	‡ 26.0 ± 1.62 (29.72%)	‡‡‡ 17.67 ± 1.82 (64.66%)	†‡‡‡ 983.57 ± 41.43 (34.25%)	†††‡‡‡ 1184.84 ± 77.27 (39.61%)

**Infected + silymarin**	‡‡‡ 25.50 ± 2.81 (49.60%)	‡‡‡ 32.17 ± 3.30 (48.61%)	‡‡‡ 19.5 ± 1.33 (47.30%)	‡‡‡ 35.5 ± 2.14 (29.00%)	†††‡ 1210.71 ± 89.85 (19.06%)	†††‡ 1628.01 ± 111.92 (17.02%)

**Infected + PZQ + silymarin**	‡‡‡§§ 8.03 ± 1.69 (84.13%)	‡‡‡§§ 20.60 ± 0.60 (67.09%)	‡‡‡§ 20.5 ± 1.14 (44.59%)	‡‡‡ 14.60 ± 1.72 (70.80%)	†‡‡‡ 964.74 ± 58.91 (35.51%)	†††‡‡‡ 1043.50 ± 58.45 (46.81%)

Data are presented as mean ± SEM (n = 8 in each group).

Mice were administered praziquantel (PZQ; 500 mg/kg/day for 2 days) in the 7^th ^week post infection (PI) and silymarin (750 mg/kg/day, 5 days/week for 6 weeks) at the 4^th ^and 12^th ^weeks PI for 6 weeks and were killed 10 and 18 weeks PI respectively. Numbers in parentheses indicate the percentage of reduction from infected (vehicle) group. TGF-β1, transforming growth factor β1; MMP-2, matrix metalloproteinase-2.

† Significantly different from uninfected (vehicle) group at *P *< 0.05 and ††† at *P *< 0.001. ‡ Significantly different from infected (vehicle) group at *P *< 0.05 and ‡‡‡ at *P *< 0.001. § Significantly different from PZQ-treated group at *P *< 0.05 and §§ at *P *< 0.01.

### Immunohistochemical aspects

**Figure 2:**
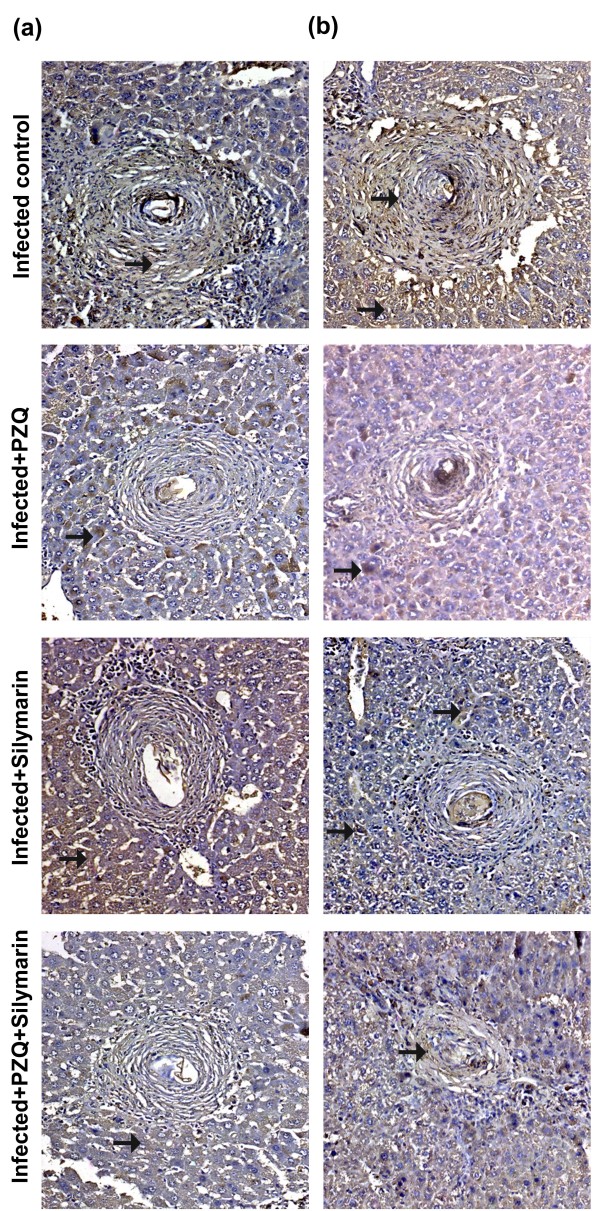
**Immunostain for TGF-β1 antibody (DAB, ×200) of infected untreated liver sections of mice killed 10 (a) and 18 (b) weeks PI showing marked positively stained hepatocytes and granuloma (fibroblast and inflammatory cells; arrows)**. Sections taken from livers of mice treated with PZQ (500 mg/kg/day for 2 days) showing mild (a) and moderate (b) positively stained hepatocytes, endothelial cells lining sinusoids and granuloma cells (arrows). Sections taken from livers of mice treated with silymarin (750 mg/kg/day, 5 days/week for 6 weeks) showing weak (a) and moderate (b) positively stained hepatocytes, endothelial cells lining sinusoids and granuloma cells (arrows). Sections taken from livers of mice treated with PZQ plus silymarin showing weakly and scattered positively stained hepatocytes and granuloma cells (a, b; arrows).

**Figure 3:**
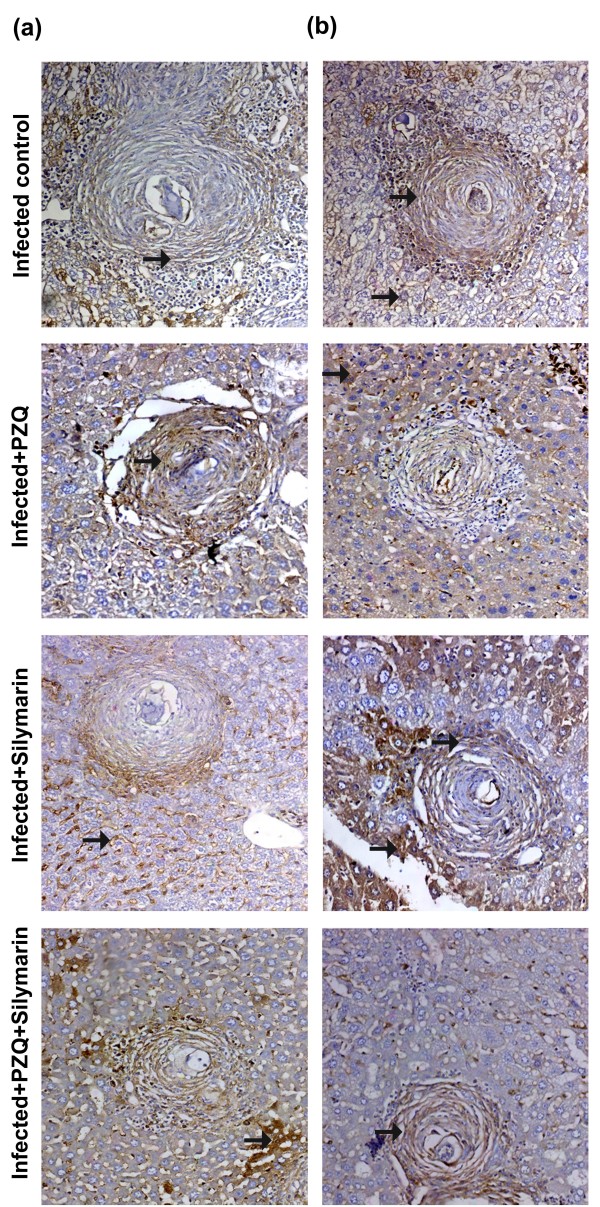
**Immunostain for MMP-2 antibody (DAB, ×200) of infected untreated liver sections of mice killed 10 (a) and 18 (b) weeks PI showing marked positively stained hepatocytes and granulomas (arrows)**. Sections taken from livers of mice treated with PZQ (500 mg/kg/day for 2 days) showing moderate (a) and mild (b) positively stained hepatocytes, endothelial cells lining sinusoids and granuloma cells (arrows). Sections taken from livers of mice treated with silymarin (750 mg/kg/day, 5 days/week for 6 weeks) showing mild (a) and moderate (b) positively stained hepatocytes, endothelial cells lining sinusoids and granuloma cells (arrows). Sections taken from livers of mice treated with PZQ plus silymarin showing scattered positively stained hepatocytes, weak positively stained endothelial cells lining sinusoids and granuloma cells (a; arrows) and mild positively stained hepatocytes, weak positively stained endothelial cells lining sinusoids and granuloma cells (b; arrows).

The control, normal, uninfected mice were negative for TGF-β1 and MMP-2 monoclonal antibodies. *S. mansoni *infected untreated mice showed positively TGF-β1 and MMP-2 stained hepatocytes (cytoplasmic), fibroblast and inflammatory cells of granulomas (Figures 2 &3a, b). Treatment with PZQ in the 7^th ^week PI significantly reduced the expression of TGF-β1 (Figure 2a & b) and MMP-2 (Figure 3a & b) by (70.75%, 52.88%; *P *< 0.001) and (29.72%; *P *< 0.05, 64.66%; *P *< 0.001) respectively, when compared with their corresponding infected untreated groups, 10 and 18 weeks PI respectively (Table 4). Treatment with silymarin in the 4^th ^and 12^th ^weeks PI significantly reduced (*P *< 0.001) the expression of TGF-β1 (Figure 2a & b) and MMP-2 (Figure 3a & b) by 49.60%, 48.61% and 47.30%, 29.00%, respectively, when compared with their corresponding infected untreated groups, 10 and 18 weeks PI, respectively. In groups treated with both PZQ and silymarin, the reduction in the expression of TGF-β1 (Figure 2a & b) was significant and reached 84.13% and 67.09% (*P *< 0.01) versus PZQ alone (70.75%, 52.88%) 10 and 18 weeks PI, respectively. Meanwhile, the reduction in the expression of MMP-2 (Figure 3a & b) was 44.59% (*P *< 0.05) and 70.80% (*P *> 0.05) versus PZQ alone (29.72%, 64.66%) 10 and 18 weeks PI, respectively (Table 4).

### Recruitment of mast cells in liver tissue

**Figure 4:**
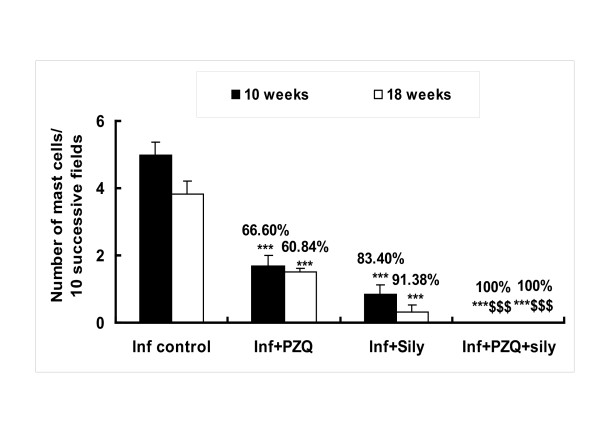
**Effect of silymarin with/without praziquantel on the total number of mast cells/10 successive fields/section, 10 and 18 weeks post-infection of mice with *S. mansoni***. Numbers in parentheses indicate the percentage of reduction from infected (vehicle) group. *** Significantly different from infected (vehicle) group at *P *< 0.001. $$$, Significantly different from infected-PZQ treated group at *P *< 0.001.

**Figure 5:**
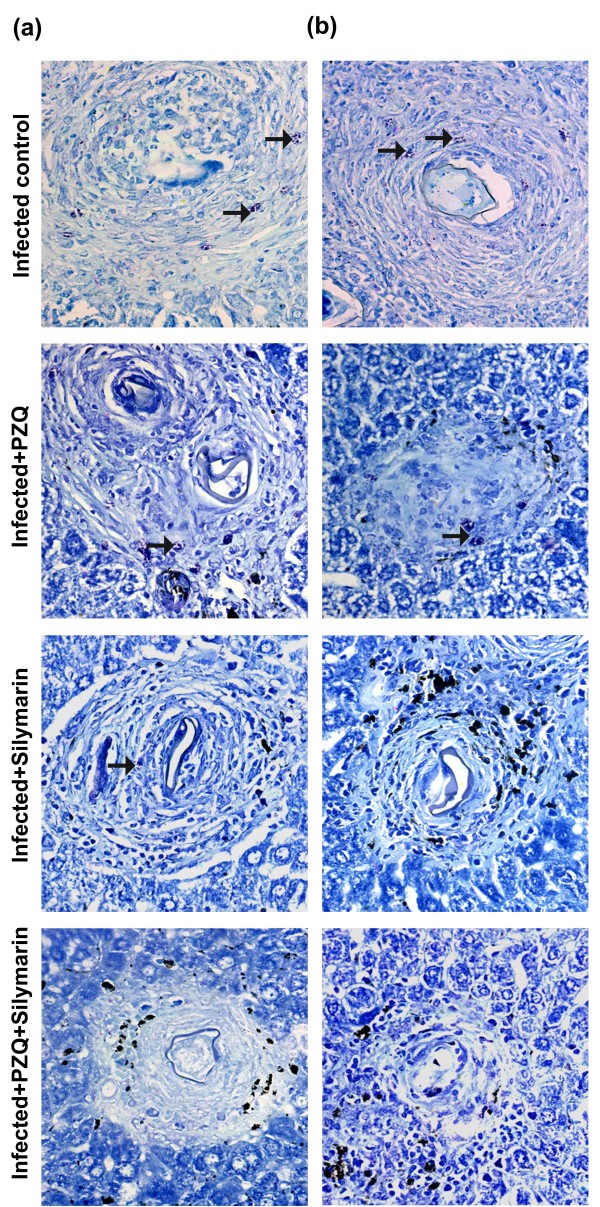
**Toludine stain (×200) of infected untreated liver sections of mice killed 10 (a) and 18 (b) wks PI showing moderate number of positively stained mast cells at granuloma sites (arrows)**. Sections taken from livers of mice treated with PZQ (500 mg/kg/day for 2 days) showing mild positively stained mast cells (a, b arrows). Sections taken from livers of mice treated with silymarin (750 mg/kg/day, 5 days/week for 6 weeks) showing few scattered mast cells (a) and scanty (b) positively stained mast cells at granuloma sites (arrows). Sections taken from livers of mice treated with PZQ plus silymarin showing complete absence of stained mast cells at granuloma sites (a) & (b).

In toluidine blue-stained liver sections from the *S.mansoni*-infected group, mast cells were oval in shape with metachromatic granules in portal areas and in the granuloma. At 10 and 18 weeks PI, the number of mast cells was 5.00 ± 0.36 and 3.83 ± 0.48/10 successive fields, respectively (Figure 4). However, in the infected PZQ-treated group, the number of mast cells was significantly decreased (*P *< 0.001) by 66.60% and 60.84%, 10 and 18 weeks PI respectively, compared with the infected untreated group. Also, in the infected silymarin-treated group, the number of mast cells was decreased significantly (*P *< 0.001) by 83.40% and 91.38% 10 and 18 weeks PI, respectively. Meanwhile, in the infected group treated with both PZQ and silymarin, complete absence of mast cells was observed 10 and 18 weeks PI, respectively (Figure 5a, b).

## Discussion

Although chemotherapy eliminates matured worms effectively and prevents the accumulation of schistosome eggs, less effective drugs are directed to reversing the existing hepatic fibrosis, especially at the chronic and advanced stages of schistosomiasis. Therefore, treatment targeting hepatic fibrosis of schistosomiasis remains a challenging proposition [31]. Because the mouse model has a similar course to human schistosomiasis [32], we, thus, established the mouse schistosomiasis model through *Schistosoma mansoni *cercariae infection, and then treated them with either silymarin alone or plus praziquantel to diminish liver fibrosis in the current study. In this study, all mice received *S. mansoni *infection in addition to the presence of hepatic granulomas around deposited schistosome eggs affecting hepatocellular function. The observed elevation in ALT reflects either acute active or chronic liver damage [33], and hypoalbuminemia in chronic infection occurs simultaneously with the increase in collagen deposition. This could be associated with malabsorption due to damaged intestinal mucosa resulting from the extrusion of large numbers of eggs, or could be due to decreased synthesis which may result from parasitic injury to hepatic cells [34]. In addition, a significant depletion of GSH, which constitutes the first line of defence against free radicals and is a critical determinant of tissue susceptibility to oxidative damage, was observed. In the present study, hydroxyproline was chosen because it is a sensitive marker that increases significantly during liver fibrosis. The increase in this amino acid reflects an increase in the *de novo *synthesis of liver collagen and an increase in the amount of hydroxyproline [35]. Here, there was a linked increase in serum MMP-2, TGF-β1 and the HYP content in both acute and chronic infections. This could be due to the activated HSCs during the acute stage of fibrosis producing more MMPs to counteract the overproduction of collagen-I, which was dominant from the onset of the fibrotic response and peaked at the chronic phase of infection [36]. Singh *et al. *[37] reported that there was a strong rise in collagen I and III expression in the liver seven weeks post infection indicating that induction of collagen production was associated with the early development of the granulomatous response. In addition, MMP-2 and TGF-β1 levels continued to rise reaching the highest level at the 18^th ^week post infection. Loebermann *et al. *[38] reported that the expression of MMP-2 correlated well with the onset and progression of fibrosis.

In the current study, treatment of *S. mansoni*-infected mice with silymarin alone at the 4^th^ and 12^th^ weeks PI resulted in remarkable worm and egg reduction accompanied with a partial increase in the percentage of dead eggs 10 and 18 weeks PI. This was associated with healing of hepatic granulomatous lesions as evidenced histopathologically with reduction of granuloma size, more granuloma circumscription, more ova degeneration and fewer inflammatory cells. This may be attributed to the antioxidative properties of silymarin [39], therefore, it is possible that silymarin eliminates the products of oxidative reactions and assists in the immune-mediated destruction of worms and eggs. Also, treatment with silymarin, whether in acute or chronic infection, significantly reduced the hepatic HYP content, tissue expression of TGF-β1 and MMP-2 and the number of mast cells. This is consistent with previous studies, which showed that administration of silymarin reduced the hepatic collagen content in diethylnitrosamine [40] and CCl_4 _administered rats [41], and exerts its anti-fibrotic properties by reducing TGF-β induced *de novo *synthesis of collagen type I [42]. Fuchs *et al. *[15] reported that prolonged treatment with silymarin resulted in its accumulation in HSCs and down regulated TGF-β1 expression; since this growth factor has the ability to induce its own production, silymarin broke the so-called fibrogenesis loop, or the self-perpetuation of hepatic fibrosis. In this study, the number of mast cells in portal areas and granulomas decreased significantly in the silymarin-treated group compared to those of the infected group. Mast cells secrete various mediators, which promote fibroblast growth, stimulate production of the extracellular matrix by fibroblasts of hepatic stellate cells, and produce components of the extramedullary matrix themselves [43]. However, it is unclear whether they play a central role in its development. Thus, silymarin has been histopathologically shown to have a significant anti-inflammatory effect on hepatic tissue, including mast cell stabilization.

In the current study, silymarin alone significantly reduced the elevated ALT and restored the depletion of GSH with no alteration in serum albumin level 10 and 18 weeks PI. Silymarin restored the elevated level of serum ALT in CCl_4 _intoxicated rats [44] and in diethylnitrosamine administered rats [45] by preventing liver damage through maintaining the integrity of the plasma membrane, thereby suppressing the leakage of enzymes. The increase in the hepatic GSH content may be attributed to the well established antioxidant actions of silymarin [39], which was able to dramatically reduce the generation of intracellular ROS in response to different pro-oxidant stimuli [42]. Furthermore, silymarin is capable of protecting liver cells directly by stabilizing the membrane permeability through inhibiting lipid peroxidation and preventing liver glutathione depletion, thus offering the synergistic benefit of sparing liver cells from destruction [46].

Reversal of schistosome-induced pathology after treatment with PZQ has been previously described [47]. In contrast to that, our findings show that PZQ alone seemed to be effective in reducing hepatic fibrosis as shown by a clear reduction in the serum levels and tissue expression of TGF-β1 and MMP-2, the number of mast cells and hepatic HYP content at 10 and 18 weeks PI. This was accompanied by a significant reduction in ALT with significant restoration of albumin and GSH. The main explanations for these results are presumed to be a removal of schistosomal worms, and subsequent reduction of egg deposition and granuloma size. Researchers showed that patients in Ethiopia and Uganda had improvement or resolution of schistosomal periportal thickening/fibrosis after parasitologic cure with PZQ [48,49]. Singh *et al. *[50] reported that with elimination of eggs and subsidence of the inflammatory response, collagen gene expression was minimal and that collagen and MMP-2 gene expression diminished in a linked fashion. The authors added that, the weakening of the inflammatory signals, may lead to a decrease in the activation of HSCs and diminished the production of IL-13 by monocytes and macrophages, which in turn reduced the stimulation of TGF-β1. In addition, in the face of decreased collagen production and deposition, expression of MMP-2 genes also decreased because the bulk of the deposited fibrous tissue had been desorbed and consequently the curtailed gene expression of MMP-2 is appropriate [50]. In a recent study by Liang *et al. *[51] on the anti-fibrotic effects of PZQ in mice with both chronic and advanced schistosomiasis as well as in CCl_4 _induced liver fibrosis mice, they mentioned that the significant amelioration of hepatic fibrosis by praziquantel treatment validates it as a promising anti-fibrosis drug and offers the potential for new chemotherapy for hepatic fibrosis resulting from schistosomiasis.

Administration of silymarin in addition to PZQ also showed complete eradication of worms, no viable eggs with reduction in the hepatic tissue egg load and more healing of hepatic granulomatous lesions revealing that the use of silymarin along with PZQ did not interfere with, or affect, the antischistosomal activity of PZQ. The highest reduction observed in TGF-β1, MMP-2 and mast cells was in the group treated with PZQ plus silymarin. In addition, the improvement of liver function and histopathology whether in acute or chronic infections may be due to blocking liver fibrosis through killing parasites and complete eradication of eggs and their toxins by PZQ to alleviate liver inflammation, and to the anti-inflammatory, anti-fibrotic actions, in addition to the antioxidative properties of silymarin. Although the hepatoprotective properties of silymarin have been reported from both *in vitro *and *in vivo *studies, its mechanism of action still has not been established [52]. Velebný *et al. *[53] showed that combined treatment with PZQ and silymarin in mice infected with *Mesocestoides vogae *(Cestoda) *tetrathyridia *was able to ameliorate or suppress fibrogenesis in the liver, protect liver cells from oxidative damage and, possibly, stimulate regeneration of the parenchyma. Interestingly, the reduction in HYP content in the group treated with silymarin plus PZQ did not reach a significant level compared with the PZQ treated group. Silymarin is thought to be dose-dependent and studies vary considerably in duration without agreement on the minimum duration needed to see the effect [54,55]. Therefore, further studies are clearly needed to investigate the efficacy of higher doses and a longer duration of treatment with silymarin in this model of liver fibrosis.

In conclusion, silymarin has partially toxic effects on worms and eggs. Furthermore, the anti-fibrotic and anti-inflammatory effects of silymarin/PZQ alone were observed in the acute and chronic fibrogenesis induced by *S. mansoni *infection. This was evident in diminishing hepatic content of HYP, serum levels and hepatic expression of TGF-β1 and MMP-2 and the number of mast cells. These effects were more obvious in most parameters studied, when silymarin was used with the removal of the causative agent by PZQ. Accordingly, our results point to silymarin as a convenient and promising therapeutic agent in the treatment of schistosomal liver fibrosis. Further studies on mechanisms of action of silymarin and praziquantel during schistosomal liver fibrosis or other chronic liver diseases may shed some light on developing therapeutic methods in clinical practice.

## Competing interests

The authors declare that they have no competing interests.

## Authors' contributions

NME, FAE and AAA conceived and designed the research; NME, WHE performed the animal experiments, parasitological and biochemical examinations; OAH and AAB performed the histological and immunohistochemical procedures; NME, WHE and OAH analyzed the data; NME interpreted data and wrote the paper. All authors read and approved the final submitted and revised versions of the manuscript.
